# Supplementary material for: Exploring structural, functional, evolutionary, and genetic characteristics of sugar transporters in maize and their roles in abiotic stress tolerance
Source: PLoS One. 2026 Feb 26;21(2):e0342990. doi: 10.1371/journal.pone.0342990 (PMC12944759; doi:10.1371/journal.pone.0342990)
Supplement: S1 Fig — (PPTX) [file pone.0342990.s010.pptx]

## Slide 1
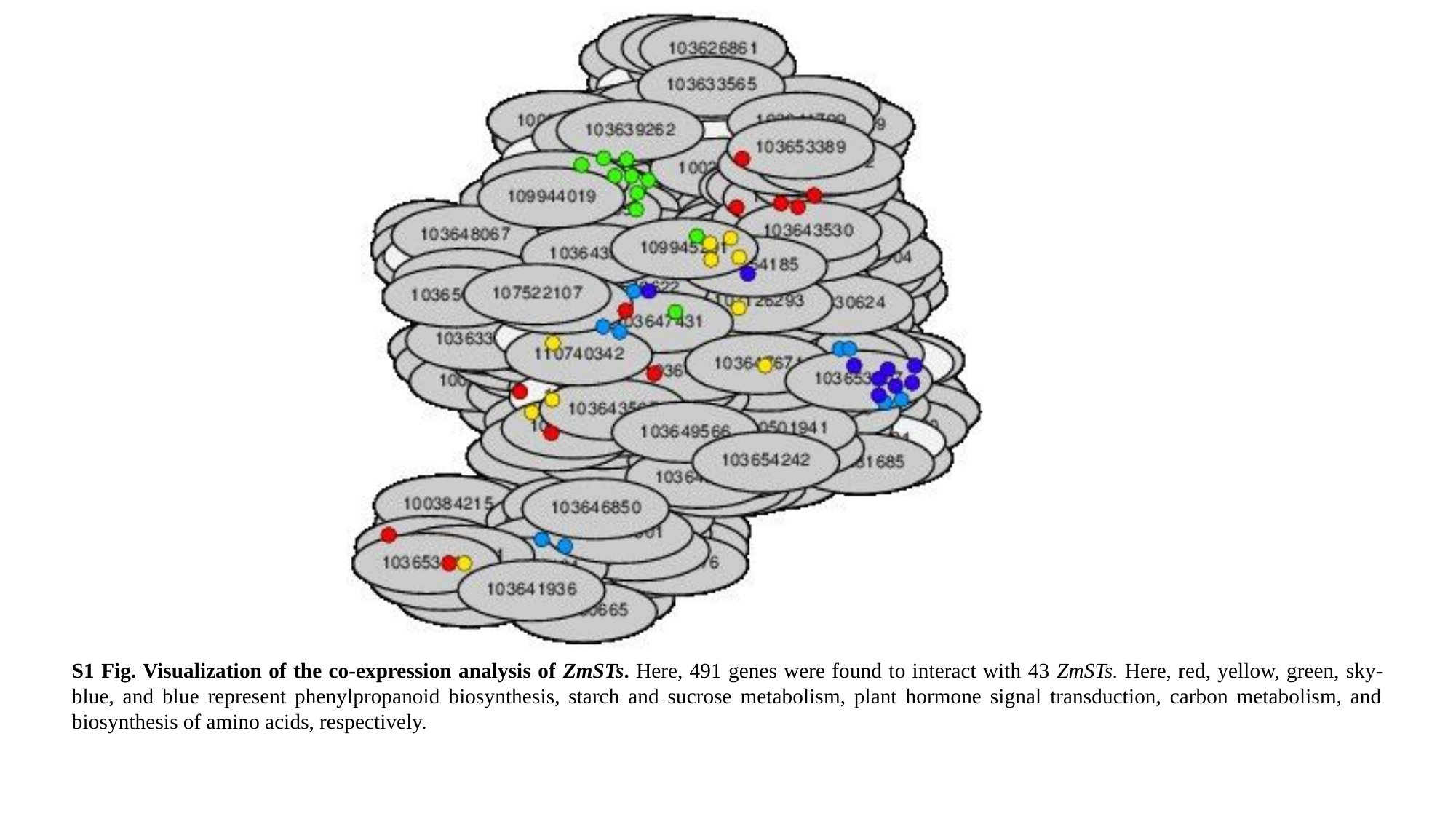

S1 Fig. Visualization of the co-expression analysis of ZmSTs. Here, 491 genes were found to interact with 43 ZmSTs. Here, red, yellow, green, sky-blue, and blue represent phenylpropanoid biosynthesis, starch and sucrose metabolism, plant hormone signal transduction, carbon metabolism, and biosynthesis of amino acids, respectively.
