# Supplementary material for: Exploring structural, functional, evolutionary, and genetic characteristics of sugar transporters in maize and their roles in abiotic stress tolerance
Source: PLoS One. 2026 Feb 26;21(2):e0342990. doi: 10.1371/journal.pone.0342990 (PMC12944759; doi:10.1371/journal.pone.0342990)
Supplement: Attachment — Submitted filename: Rebuttal Letter__R1.docx [file pone.0342990.s012.docx]

We sincerely appreciate the reviewers for their thorough evaluation and insightful comments, which have significantly improved the quality and scientific rigor of our manuscript. All revisions are done under track change option. In the revised version, all changes made in response to **Reviewer 1** are highlighted in yellow, and the same for **Reviewer 2** are highlighted in green for clarity and transparency. Below, we provide a detailed, point-by-point response to each comment raised by the reviewers. Additional revisions are under non-highlighted track change option.

In addition to revised version under track change option, we are also adding clean version of the manuscript.

**Editorial office comment**

**Comment 1:** Please include a figure label and title for Figure 1 to 13 in your main manuscript.

**Response:** Thank you for your comment. In revision, we have added the figure label to our revised manuscript. Please see the revised manuscript. Lines 986-1080 in the clean version and lines 1631-1723 in the track change version manuscript.

**Response to the Journal requirements**

**Journal Requirements:**

When submitting your revision, we need you to address these additional requirements.

**Comment 1:** Please ensure that your manuscript meets PLOS ONE's style requirements, including those for file naming. The PLOS ONE style templates can be found at

https://journals.plos.org/plosone/s/file?id=wjVg/PLOSOne_formatting_sample_main_body.pdf and

<https://journals.plos.org/plosone/s/file?id=ba62/PLOSOne_formatting_sample_title_authors_affiliations.pdf>

**Response:** Thank you for your helpful comment. We have reviewed the PLOS ONE style guidelines, and we will ensure that the manuscript adheres to the required formatting, including file naming. We will follow the provided templates for both the manuscript body and the title/author affiliations section. The revised version of the manuscript will be submitted in full compliance with these formatting requirements.

**Comment 2:** Please note that PLOS ONE has specific guidelines on code sharing for submissions in which author-generated code underpins the findings in the manuscript. In these cases, we expect all author-generated code to be made available without restrictions upon publication of the work. Please review our guidelines at https://journals.plos.org/plosone/s/materials-and-software-sharing#loc-sharing-code and ensure that your code is shared in a way that follows best practice and facilitates reproducibility and reuse.

**Response:** Thank you for the comment. We would like to clarify that no author-generated or custom code was developed or used in this study. Therefore, the code-sharing policy is not applicable to our manuscript. All analyses were conducted using standard, widely available tools, and the methodology has been described in sufficient detail to ensure reproducibility.

**Comment 3:** We note that the grant information you provided in the ‘Funding Information’ and ‘Financial Disclosure’ sections do not match.

When you resubmit, please ensure that you provide the correct grant numbers for the awards you received for your study in the ‘Funding Information’ section.

**Response:** Thank you for your comment. We have carefully reviewed the ‘Funding Information’ and ‘Financial Disclosure’ sections and corrected them to ensure that the grant numbers and funding details are accurate and consistent across both sections.

**Comment 4:** Thank you for stating the following financial disclosure:

“The Natural Science Foundation of Sichuan Province (Grant No. 2022NSFSC1774), the National Natural Science Foundation of China (Grant No. 32101673), the Liangshan Prefecture Science and Technology Program (24YYYJ0184 and 24YYYJ0183), the Sichuan Science and Technology Program (Grant No. 2021YFYZ0017), and the Academician Expert Workstation of Yunnan Province (to Han Lan, Grant No. 202305AF150052) provided support for this work. The Yunnan Major special research project "Creation and application of special bio-fertilizer for Plateau characteristic economic crops in Yunnan Province" (202202AE090015) also provided money for the work. King Saud University in Riyadh, Saudi Arabia, (project number RSP2026R357) for providing technical support of molecular dynamics simulation experiment.”

Please state what role the funders took in the study.  If the funders had no role, please state: "The funders had no role in study design, data collection and analysis, decision to publish, or preparation of the manuscript."

If this statement is not correct you must amend it as needed.

Please include this amended Role of Funder statement in your cover letter; we will change the online submission form on your behalf.

**Response:** Thank you for the comment. We would like to clarify that the funders were involved in specific aspects of the study. In particular, the funders contributed to the study design, formal analysis, technical support, and supervision of the research. The manuscript and cover letter have been amended to include the following statement:

“The funders were involved in study design, formal analysis, technical support, and supervision of the research. They had no role in data collection, decision to publish, or preparation of the manuscript.”

Please include our current funding status. Our corrected funding’s status is-

“The Natural Science Foundation of Sichuan Province (Grant No. 2022NSFSC1774), the National Natural Science Foundation of China (Grant No. 32101673), the Liangshan Prefecture Science and Technology Program (24YYYJ0184 and 24YYYJ0183), the Sichuan Science and Technology Program (Grant No. 2021YFYZ0017), and the Academician Expert Workstation of Yunnan Province (to Han Lan, Grant No. 202305AF150052) provided support for this work. The Yunnan Major special research project "Creation and application of special bio-fertilizer for Plateau characteristic economic crops in Yunnan Province" (202202AE090015) also provided money for the work. King Saud University in Riyadh, Saudi Arabia, (project number ORF-2026-357) for providing technical support of molecular dynamics simulation experiment.”

We appreciate the opportunity to clarify this information.

**Comment 5:** Please note that funding information should not appear in any section or other areas of your manuscript. We will only publish funding information present in the Funding Statement section of the online submission form. Please remove any funding-related text from the manuscript.

**Response:** Thank you for the comment. We have revised according to your guidance. Please see the revised manuscript.

**Comment 6:** "Please include a caption for figure 6.

**Response:** Thank you for the comment. Revised accordingly. Please see the revised figure.

**Comment 7:** Please include captions for your Supporting Information files at the end of your manuscript, and update any in-text citations to match accordingly. Please see our Supporting Information guidelines for more information: <http://journals.plos.org/plosone/s/supporting-information>.

**Response:** Thank you for the comment. Revised accordingly. Please see the revised manuscript.

**Comment 8:** If the reviewer comments include a recommendation to cite specific previously published works, please review and evaluate these publications to determine whether they are relevant and should be cited. There is no requirement to cite these works unless the editor has indicated otherwise.

**Response:** Thank you for the comment.

[Note: HTML markup is below. Please do not edit.]

**Reviewers' comments:**

Reviewer's Responses to Questions

**Comments to the Author**

1. Is the manuscript technically sound, and do the data support the conclusions?

The manuscript must describe a technically sound piece of scientific research with data that supports the conclusions. Experiments must have been conducted rigorously, with appropriate controls, replication, and sample sizes. The conclusions must be drawn appropriately based on the data presented.

Reviewer #1: Partly

Reviewer #2: Partly

**Response:** We thank both reviewers for their careful evaluation of our manuscript. In response to their comments, we have thoroughly revised the manuscript to improve its technical soundness and to better demonstrate that the data support the conclusions. Specifically, we have clarified the experimental design, strengthened the description of controls and replication, and revised the Results and Discussion sections to ensure that all conclusions are directly supported by the presented data.

2. Has the statistical analysis been performed appropriately and rigorously?

Reviewer #1: N/A

Reviewer #2: No

**Response:** We thank the reviewer for this important comment. In response, we have carefully revised the statistical analysis to improve its rigor and clarity.

3. Have the authors made all data underlying the findings in their manuscript fully available?

The [PLOS Data policy](http://www.plosone.org/static/policies.action#sharing) requires authors to make all data underlying the findings described in their manuscript fully available without restriction, with rare exception (please refer to the Data Availability Statement in the manuscript PDF file). The data should be provided as part of the manuscript or its supporting information, or deposited to a public repository. For example, in addition to summary statistics, the data points behind means, medians and variance measures should be available. If there are restrictions on publicly sharing data—e.g. participant privacy or use of data from a third party—those must be specified.

Reviewer #1: Yes

Reviewer #2: No

**Response:** Thank you for the comment. We confirm that all data underlying the findings of this study have been made fully available without restriction in the revised manuscript.

4. Is the manuscript presented in an intelligible fashion and written in standard English?

PLOS ONE does not copyedit accepted manuscripts, so the language in submitted articles must be clear, correct, and unambiguous. Any typographical or grammatical errors should be corrected at revision, so please note any specific errors here.

Reviewer #1: No

Reviewer #2: No

**Response:** Thank you for the comment. The manuscript has been thoroughly revised to improve readability, clarity, and consistency with standard English. Grammatical errors, typographical issues, and ambiguous expressions have been corrected throughout the text.

5. Review Comments to the Author

Please use the space provided to explain your answers to the questions above. You may also include additional comments for the author, including concerns about dual publication, research ethics, or publication ethics. (Please upload your review as an attachment if it exceeds 20,000 characters)

**Response to Reviewer 1**

Reviewer #1: This study represents a meaningful investigation into maize sugar transporters, which play crucial roles in transmembrane sugar transport and tissue-specific functions. The authors systematically identified eight subclasses of sugar transporters in maize, analyzed their sequence characteristics, and conducted expression profiling of ZmPLT1, ZmPLT8, ZmSTP1, ZmTMT1, and ZmSUC3 under various conditions. The membrane localization of ZmPLT1 and ZmPLT8 was experimentally validated. While the work provides foundational insights, substantial improvements are required for publication.

**Comment 1:** The manuscript requires thorough linguistic editing to resolve ambiguous phrasing. Notable examples include:
Abstract: "Co-expression of maize sugar transporter genes was co-expressed with stress responsive transcription factors MYB8-ZmSTP9 and A6b-ZmPLT10..." (ambiguous causal linkage)
Abstract: "After ZmPLT1, 8, ZmSTP1, ZmTMT1, and ZmSUC3 genes were validated by real-time PCR..." (redundant structure).

**Response:** Thank you for the comment. We have revised the highlighted sentences to improve clarity, remove redundancy, and avoid unintended causal implications. Please see the revised manuscript.

2. Line 49, Do the designations 'MYB8-ZmSTP9' and 'A6b-ZmPLT10' conform to the nomenclature conventions for transcription factors?

**Response:** Thank you for the comment. We clarify that the terms “MYB8–ZmSTP9” and “A6b–ZmPLT10” were not intended to represent official transcription factor names. Rather, they were used to denote co-expression relationships between the transcription factors (MYB8 and A6b) and their associated target genes (*ZmSTP9* and *ZmPLT10*), respectively. Please see the revised manuscript. Lines 57-63 in the clean version and lines 80-83 in the track change version.

3. Line 50, “8” should revised to “ZmPLT8”.

**Response:** Thank you for the comment. Revised accordingly, please see the revised manuscript. Line 62 in the clean version and line 89 in the track change version.

4. Line 57, Reassess keyword selection to reflect core findings.

**Response:** Thank you for the comment. Revised accordingly, please see the revised manuscript. Lines 71-72 in the clean version and lines 106-108 in the track change version.

5. Line 58, Use standardized abbreviations: "qRT-PCR", "mL" (throughout).

**Response:** Thank you for the comment. Revised accordingly, please see the revised manuscript. Lines 214 and 382 in the clean and track change version, respectively.

6. Line 127, Ensure binomial nomenclature formatting (italicize Zea mays L. with roman "L").

**Response:** Thank you for the comment. Revised accordingly, please see the revised manuscript. Line 101 and 199 in the clean and track change version, respectively.

7. Line 150, Specify database versions/access dates for PFAM, CDD, and SMART analyses.

**Response:** Thank you for the comment. Revised accordingly, please see the revised manuscript. Line 123 and 250 in the clean and track change version, respectively.

8. Line 174, Report bootstrap values supporting phylogenetic tree topology.

**Response:** We thank the reviewer for this comment. In the present study, the phylogenetic analysis was primarily conducted to classify maize, rice, and Arabidopsis sugar transporter proteins into distinct clades and to illustrate their overall evolutionary relationships. The major clades were consistently resolved and well supported by the maximum likelihood approach based on established sequence similarity and domain composition. Line 149 and 276 in the clean and track change version, respectively.

Given that the phylogenetic tree was not intended to infer fine-scale evolutionary divergence but rather to support gene family classification, bootstrap values were not included in the current analysis. We have clarified this purpose in the revised manuscript to avoid overinterpretation of the tree topology.

9. Lines 245-248: Create dedicated paragraph for materials description.

**Response:** Thank you for the comment. We have revised the manuscript to present the materials description in a dedicated paragraph. The plant material, growth conditions, sampling location, and sampling time points are now clearly described in a separate paragraph in the Materials and Methods section to improve clarity and organization. Lines 232-237 in the clean version and lines 365-370 in the track change version.

10. Lines 300-304, Provide evolutionary significance rather than statistical counts.

**Response:** Thank you for the comment. Revised accordingly, please see the revised manuscript. Lines 304-313 in the clean version and lines 449-463 in the track change version.

11. Lines 336-339, Re-evaluate conclusions drawn from synteny analysis.

**Response:** Thank you for the comment. Revised the manuscript accordingly. Please see the revised manuscript. Lines 352-357 in the clean version and lines 534-540 in the track change version.

12. Line 487, Distinguish co-expression networks from protein-protein interactions.

**Response:** Thank you for the comment. In the revised version, co-expression results are now explicitly described as indicators of potential functional association rather than physical interaction. Please see the revised manuscript. Line 488 and 803 in the clean and track change version, respectively.

13. Lines 281-285, Resolve numerical discrepancy: 59 vs. 60 reported genes.

**Response:** Thank you for the comment. Upon careful re-examination, we corrected the discrepancy in the reported number of genes to ensure consistency throughout the manuscript. The revised version now consistently reports the correct total number of genes, and all relevant sections, tables, and figures have been updated accordingly. Line 281 and 422 in the clean and revised version, respectively.

14. Section 3.7: Remove redundant subheading hierarchy. Streamline bioinformatics narratives to enhance readability.

**Response:** Thank you for the comment. Revised accordingly, please see the revised manuscript. Line 414 and 644 in the clean and track change version, respectively.

15. There are no bootstrap values in phylogenetic trees.

**Response:** Thank you for the comment. The phylogenetic analysis was performed to classify sugar transporter genes into major clades and to provide a comparative overview across species, rather than to evaluate branching confidence or infer detailed evolutionary relationships. Therefore, bootstrap values were not included. The scope of the phylogenetic analysis has been clearly stated in the revised manuscript to prevent overinterpretation.

16. Methods: Describe protein interaction prediction methodology (missing for Fig. 5).
**Response:** Thank you for the comment. Revised and clearly stated in the Method section, please see the revised manuscript. Lines 185-191 in the clean version and lines 313-319 in the track change version.

17. Fig. 5: Clarify methodology for protein interaction predictions.

**Response:** Thank you for the comment. Revised and clarified in the Method section, please see the revised manuscript. Lines 186-188 in the clean version and lines 314-316 in the track change version.

18. Fig. 10: Replace numerical labels with interpretable biological annotations.

**Response:** Thank you for the comment. Revised accordingly, please see the revised manuscript.

19. Fig. 13: Justify the "nucleus" designation in localization results, but the result is not correct.

**Response:** Thank you for the comment. The “nucleus” label in Fig. 13 was an annotation error. The description of figure has now been corrected, and the localization is consistently labeled as plasma membrane, which accurately reflects the experimental results. The figure legend and corresponding text have been updated accordingly.

20. Line 1082: Cite English-language version when available.

**Response:** Thank you for the comment. Revised accordingly. Please see the revised manuscript.

While the study contains valuable data, substantial reorganization and methodological clarifications are required to strengthen scientific rigor and narrative coherence. A focused revision addressing these concerns would significantly enhance the manuscript's impact.

**Response:** We thank the reviewer for this constructive assessment. In response, we have substantially revised and reorganized the manuscript, clarified methodological details, and improved narrative coherence. These focused revisions have strengthened the scientific rigor and overall impact of the study.

**Response to Reviewer 2**

Reviewer #2: I have reviewed the manuscript entitled “Exploring structural, functional, evolutionary, and genetic characteristics of sugar transporters in maize and their roles in abiotic stress tolerance” (PONE-D-25-21914), which presents a comprehensive in silico and experimental survey of sugar transporter gene families in maize, integrating phylogenetics, structural prediction, synteny, docking and molecular dynamics simulations, expression profiling, qRT-PCR validation, and subcellular localization. While the topic is relevant and the dataset assembled is extensive, the manuscript in its current form raises significant concerns regarding novelty, analytical depth, and suitability for publication in PLOS ONE. The study largely follows a well-established “genome-wide identification plus expression analysis” template that has been repeatedly applied to transporter and transcription factor families across many plant species, and the present work does not clearly articulate a conceptual or methodological advance beyond incremental expansion of descriptive analyses. The molecular docking and MD simulations, although technically executed, are not convincingly linked to biological function or experimental validation, and their inclusion appears speculative, with limited interpretive value for in vivo sugar transport or stress tolerance mechanisms. Similarly, the expression analyses under abiotic stresses are predominantly descriptive, lacking statistical testing, functional perturbation (e.g., mutant or overexpression lines), or causal evidence connecting specific transporters to stress tolerance phenotypes. The manuscript is also excessively long and diffuse, with extensive repetition of background information and methodological detail that obscures the central findings and weakens the overall narrative. Although the authors state that all relevant data are available within the manuscript and supplementary files, clearer organization and stronger synthesis would be required to make the work accessible to a broad readership. Overall, despite the breadth of analyses presented, the study does not sufficiently meet the standards of novelty, mechanistic insight, or analytical rigor expected for a general-interest journal such as PLOS ONE, and I therefore recommend rejection or, at minimum, major revision with a substantially strengthened focus on biological significance, reduced redundancy, and clearer justification of the study’s contribution beyond descriptive genomics.

**Response:** Thank you for your thoughtful and constructive feedback on our manuscript. We appreciate the recognition of the relevance of our study, and we have taken all suggestions seriously to improve the manuscript. Regarding the concern about novelty, we acknowledge that the study employs well-established methodologies; however, we have clarified in the introduction and discussion how our integrated approach of combining bioinformatics prediction, molecular dynamics simulation, and experimental validation advances beyond the traditional genome-wide identification and expression analysis. We have strengthened the analytical depth by revisiting the molecular docking and MD simulation, add statistical parameters in methods and materials section, providing a more detailed explanation of their biological relevance to stress tolerance. Our results expand the understanding of how ligand binding and hydrogen bonding at the molecular level may regulate sugar transport in maize, providing new targets for genetic improvement and stress tolerance research. To address the concern about suitability for PLOS ONE, we have placed greater emphasis on the biological significance and mechanistic insights derived from our approach, underscoring the broader implications for plant stress tolerance. We also agree with the comment about the manuscript length and redundancy, and we have streamlined the content by condensing background information and methodological details, ensuring the central findings are clearer and more concise. Lastly, we have explicitly highlighted the contribution of our study beyond descriptive genomics, discussing how our integrative approach could inform future research on functional genomics and stress tolerance mechanisms. We believe that the revisions made in response to your valuable feedback have strengthened the manuscript and brought it closer to meeting the standards of PLOS ONE. Thank you again for your insightful comments, which have greatly improved the quality of our work.

6. PLOS authors have the option to publish the peer review history of their article ([what does this mean?](https://journals.plos.org/plosone/s/editorial-and-peer-review-process#loc-peer-review-history)). If published, this will include your full peer review and any attached files.

If you choose “no”, your identity will remain anonymous but your review may still be made public.

**Do you want your identity to be public for this peer review?** For information about this choice, including consent withdrawal, please see our [Privacy Policy](https://www.plos.org/privacy-policy).

Reviewer #1: No

Reviewer #2: No

**Response:** Thank you for the comment.

[NOTE: If reviewer comments were submitted as an attachment file, they will be attached to this email and accessible via the submission site. Please log into your account, locate the manuscript record, and check for the action link "View Attachments". If this link does not appear, there are no attachment files.]

To ensure your figures meet our technical requirements, please review our figure guidelines: https://journals.plos.org/plosone/s/figures

You may also use PLOS’s free figure tool, NAAS, to help you prepare publication quality figures: https://journals.plos.org/plosone/s/figures#loc-tools-for-figure-preparation.

NAAS will assess whether your figures meet our technical requirements by comparing each figure against our figure specifications.

**Response:** Thank you for the comment. The figures are revised and updated accordingly. Please see the revised figures.

*In compliance with data protection regulations, you may request that we remove your personal registration details at any time.* [*(Remove my information/details)*](https://www.editorialmanager.com/pone/login.asp?a=r)*. Please contact the publication office if you have any questions.*

We are grateful to the reviewer for constructive suggestions, which have significantly improved the clarity and depth of our manuscript.

Sincerely,

Corresponding Author on behalf of all co-authors
